# Immunity and Tolerance Induced by Intestinal Mucosal Dendritic Cells

**DOI:** 10.1155/2016/3104727

**Published:** 2016-02-29

**Authors:** Julio Aliberti

**Affiliations:** Division of Immunobiology, Cincinnati Children's Hospital Medical Center, Cincinnati, OH 45229, USA

## Abstract

Dendritic cells present in the digestive tract are constantly exposed to environmental antigens, commensal flora, and invading pathogens. Under steady-state conditions, these cells have high tolerogenic potential, triggering differentiation of regulatory T cells to protect the host from unwanted proinflammatory immune responses to innocuous antigens or commensals. On the other hand, these cells must discriminate between commensal flora and invading pathogens and mount powerful immune response against pathogens. A potential result of unbalanced tolerogenic versus proinflammatory responses mediated by dendritic cells is associated with chronic inflammatory conditions, such as Crohn's disease, ulcerative colitis, food allergies, and celiac disease. Herein, we review the dendritic cell population involved in mediating tolerance and immunity in mucosal surfaces, the progress in unveiling their development in vivo, and factors that can influence their functions.

## 1. Introduction

The digestive tract is in direct contact with foreign antigens and microorganisms. The ability of the immune system to keep tolerance to commensals while remaining capable of responding to injury or infection with pathogenic microorganisms is essential for tissue homeostasis. Any disturbances in this balance either by genetic, environmental, or infectious causes can lead to chronic inflammatory and/or autoimmune diseases. The mucosal immune system should sense pathogens versus innocuous dietary antigens or commensal microorganisms. While a strong and protective response is required to eliminate pathogens, tolerance is essential for harmless antigens or nutrients, thus avoiding inflammatory responses.

During oral tolerance systemic immune effector function including delayed type hypersensitivity response and IgE antibody production are affected [1, 2]. Furthermore, intestine-resident effector cells also undergo tolerance. Impairment of oral tolerance seems to be associated with coeliac disease, characterized by an aberrant Th1-mediated DTH triggered by dietary gluten [1, 3]. Similarly, IgE-mediated food allergies can be derived from the break of tolerance to food antigens [1, 4].

Along the same lines, break of tolerance at the large intestine is thought to trigger hyperreactivity to commensal bacteria resulting in inflammatory bowel diseases, including Crohn's disease [5]. Interestingly, tolerance to commensal flora does not exert a systemic effect [6, 7]. Moreover, IgA production is maintained, thus supporting commensalism, because of the noninflammatory properties of IgA [8, 9].

The induction of oral tolerance has been the object of several studies. It is well accepted that clonal deletion and/or T cell anergy are components of the mechanism of action of oral tolerance, however induction of regulatory T cells (Treg's) has become widely known as its central component [10]. The induction of FoxP3+ Treg cells requires CD103+ dendritic cells (DCs). Herein, we will review the development/differentiation of mucosal resident DC subsets and their relative contribution to tolerance and immunity.

## 2. Subsets and Function

Intestinal DCs are located throughout the villus lamina propria and in intestinal lymphoid tissue (Peyer's Patches, solitary isolated lymphoid tissue, and mesenteric LN), where they play a central role in sampling and processing luminal as well as peripheral self-antigen for presentation to T cells [10]. A seminal study by Rescigno et al. [11] showed that CD11c+ cells send transepithelial dendrites from the lamina propria that penetrate through tight junctions and capture* Salmonella* from the lumen. Lamina propria contains two major populations of CD11c+ mononuclear phagocytes: CD11c^hi^CD103+CD11b+CX3CR1− cells (DCs) and CD11c^int^CD103−CD11b+CX3CR1+ cells (macrophages) [6, 9, 12–15]. CX3CR1+ macrophages, rather than the CD103+ DCs, are sampling the intestinal luminal content by extending transepithelial dendrites [11, 13, 16–18]. Exposure to TLR-ligands [13] and microbes [18] induces transepithelial dendrites formation [17]. CD103+ DCs have not been observed extending transepithelial dendrites [17].

**Figure 1:**
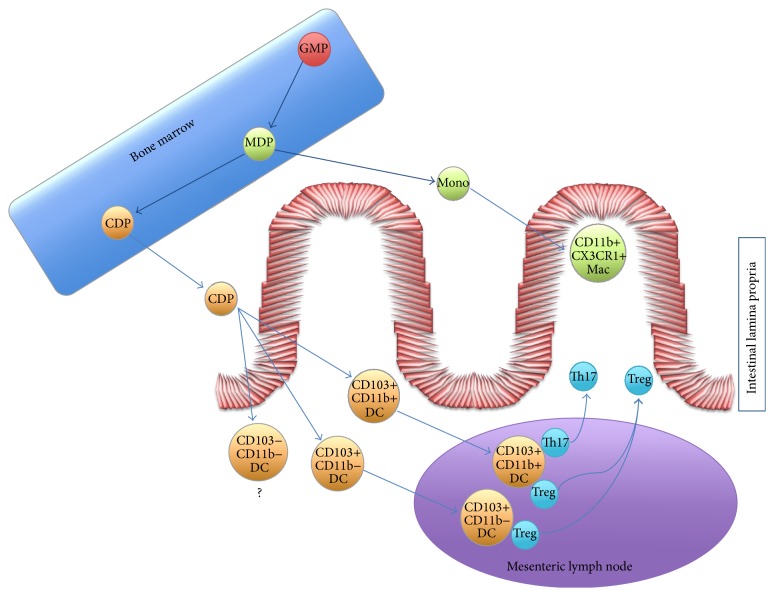
Intestinal mucosal dendritic cell and macrophage development and function. Bone marrow resident Granulocyte Macrophage Progenitors (GMP) give rise to Macrophage DC Precursors (MDP). In turn, CDP give rise to peripheral blood monocytes (Mono) and Common DC Progenitors (CDP). Monocytes will migrate to the lamina propria differentiating into CD11b+CX3CR1+ macrophages that directly sample antigens from the intestinal lumen. On the other hand, CDP will give rise to three subpopulations of intestinal lamina propria DCs: CD11b+CD103+, CD11b−CD103+, and CD11b−CD103−. The former two subsets are responsible for sampling antigen and priming naïve T cells into regulatory T cells (Treg) or IL17-producing T cells (Th17).

DCs (CD11c+CX3CR1− cells) can be further subdivided into three major subsets based on the expression of CD11b and CD103, with CD11b+CD103+, CD11b−CD103+, and CD11b−CD103− [19, 20] (Figure 1). Lymphoid tissue resident DCs include plasmacytoid DCs (pDCs) and CD8*α*+ and CD11b+ conventional DCs (cDCs). They can be found along the lymphoid organs associated with the intestine, including PPs, isolated lymphoid follicles, and MLNs. Nonlymphoid tissue DCs, found in the parenchyma of tissues, are also known as migratory DCs. Under steady-state conditions, migratory DCs promote the expansion of regulatory T cells, required for tolerance to self-antigens [21, 22] (Figure 1). On the other hand, during inflammatory response to infection, these cells promote protective T cell responses [23, 24]. The expression of the chemokine receptor CCR7 and its ligands CCL21 and CCL19 control whether migratory DCs move into draining LNs [25].

Classic process of DC maturation occurs upon exposure to microbial stimuli or proinflammatory cytokines. Typically, morphological, phenotypic, and functional changes are observed. Such modifications are essential for effective naïve T cells priming and activation. On the other hand, migratory DC maturation is associated with tolerance induction rather than activation and proliferation, despite upregulation in MHC II and CD40 [20]. Importantly, the signals that trigger and modulate such maturation processes are poorly understood.

Induction of tolerance versus immunity by intestinal DC is, at least in part, mediated by retinoic acid receptors (RAR) signaling [26–29]. Thus, exposure to RA triggers expression of gut-homing receptors along with enhancing expansion of FoxP3+ T cell and IgA B cell differentiation. On the other hand, antagonists of RAR inhibit expansion of such cells [30–32]. Induction of gut-homing receptors on primed T cells as well as FoxP3+ T cell differentiation in vitro is best achieved in the presence of migratory (CD103+CD11b− or CD11b+) DCs among other DC subsets [4, 5, 7]. RALDH2 is one of the enzymes that metabolize retinal to RA, CD103+ DCs express high levels of the gene encoding it –* aldh1a2*. Consistently, CD103+ DCs triggered RAR-dependent signaling in responding T cells [33]. Small intestine-lamina propria and MLN resident CD103+ DCs trigger RAR signals and induce expression of CCR9 in responding T cells [34]. All DCs trigger limited RAR signaling in T cells; however high levels of CCR9 induction are a key function associated with small intestine-lamina propria and MLN CD103+ DCs. On the other hand, the CD103+CD11b+ subset seems critical for the induction of proinflammatory Th17 cells [19, 20] given its high induction of IL6 in response to microbial stimulation [35].

## 3. Mucosal Dendritic Cell Precursors and Homing Markers

The interaction of FMS-like tyrosine kinase 3 (Flt3) with its ligand (Flt3-L) is critical for the generation of CD103+ DCs [36], both in mice and humans [37, 38]. Pre-B cells as well as myeloid and monocytic lineages show upregulated Flt3 mRNA, while Flt3-L mRNA expression is ubiquitous [39]. Both Flt3 and Flt3-L show high conservation in mice and humans. Treatment of mice with human Flt3-L leads to activation of mouse Flt3 [40] triggering bone marrow hyperplasia along with hematopoietic stem and progenitor cell proliferation. Interestingly, FLT3-L showed a positive bias in the expansion of CD103+ DCs [13].

Macrophage and DC bone marrow precursors give rise to monocytes and common DC progenitor [41]. Common DC progenitors are comprised within lineage (lin)- negative, Flt3-L+ cell subset [42, 43] (Figure 1). PDCs and cDCs are both derived from the common DC precursor within this lin- Flt3+ compartment [44, 45]. The common DC precursor is GM-CSF receptor *α*+ [45]. The transcription factor IRF8 is required for development and activation of pDCs and CD8*α*+ DCs [46–48] and PU.1 is important for all conventional (nonplasmacytoid) DCs [49, 50]. The expression of PU.1 is induced by Flt3 signaling [51]. Intestinal CD103+CD11b− DCs are developmentally related to the CD8*α*+ lymphoid DC subset, since both subsets are dependent on the presence of the transcription factors IRF8, Id2, and BATF3 [52].

Most CD103+ small intestine-lamina propria DCs have been shown to develop directly from a circulating FLT3+ common DC precursor and not from CD103− small intestine-lamina propria DCs [53] (Figure 1). Interestingly, a great proportion of MLN resident CD103+ DCs are thought to be derived from a migratory population arriving from small intestine-lamina propria that plays a critical role in presenting orally derived soluble antigen to T cells (Figure 1). Presumably, these cells seize antigens locally in the small intestine and subsequently migrate into the MLN. On the other hand, CD103− MLN DCs appear to be derived from a blood population that populate and expand the MLN and is involved in the T cell priming to systemic antigens [53]. Importantly, CD103+ DCs are present in normal and inflamed human MLN and display similar phenotypic and functional properties to their murine counterparts [6].

CCR7, a chemokine receptor which is required for DC migration from peripheral tissues into the draining LN, is required for accumulation of CD103+ DC in the MLN, as CCR7-deficient hosts have reduced numbers of MLN CD103+ DCs [54–56].

## 4. Intestinal Mucosal Dendritic Cell Responses to Infection

Intestinal flora is composed of trillions of resident bacteria that can provide beneficial effects to the host [57]. For example, bacterial metabolites including vitamins and short chain fatty acids are relevant for the host development, including lymphoid populations in the intestine. Moreover, resident bacteria mediate resistance against pathogen infection [58]. Several host immune-regulatory mechanisms have evolved to prevent inappropriate activation of inflammatory responses in response to the commensal flora, including the hyporesponsiveness of intestinal epithelium and resident macrophages to bacterial Toll-like receptor ligands [59, 60]. However, intestinal microbiota can potentially trigger (or enhance) an inflammatory response. Chemically induced and spontaneous colitis are reduced or abolished in antibiotic-treated mice and germ-free mice [61–65] and* Bacteroides* species and members of the Enterobacteriaceae family including* Klebsiella pneumoniae* and* Proteus mirabilis* can promote colitis [66, 67].

Activation of inflammatory responses by flora is mediated by host pattern-recognition receptors [68]. Inflammasome, a multiprotein complex that leads to caspase-1 initiated proteolytic processing of pro-interleukin-1*β* and pro-IL18 into their active forms [69]. In the intestine,* Salmonella* triggers resident phagocytes to produce IL-1*β* in an NLRC4-dependent manner leading to neutrophil recruitment [70].

The role of the NLRP3 inflammasome in intestinal inflammation is controversial. On one hand, mice lacking NLRP3 or caspase-1 were shown to be less susceptible to chemically induced colitis [71, 72]. On the other hand, it was shown that these same animals had increased susceptibility and worsened pathology [73, 74]. Along the same lines, the role of IL-1*β* in colitis is also controversial. While IL-1*β* blockage improves intestinal inflammation in different animal colitis models [75, 76], another study showed that genetic deficiency of IL-1*β* leads to increased susceptibility to experimental colitis [8]. Although it is not clear what the reasons for such differences in results are, one potential explanation is the composition of gut flora [71]. For instance,* Escherichia coli* trigger NLRP3 inflammasome in bone marrow derived macrophages to produce IL-1*β* [77, 78].

## 5. Mucosal Tolerance and Dendritic Cells

Several commensal* Bacteroides* and Bifidobacteria strains can directly induce monocyte-derived DCs to acquire a tolerogenic phenotype [79]. Polysaccharide A from* Bacteroides fragilis*, a Gram-negative anaerobic commensal bacterium, can also associate with CD11c+ cells in MLNs and drive a mixture of Th1 systemic responses and IL10-producing Treg cells in the colonic LP [80]. Segmented filamentous bacteria induce differentiation of both mucosal Th17 and FoxP3+ Treg cell. These effects are associated with the modulation of APC function in the lamina propria [19, 81, 82]. Antigen presentation by CD103+ DCs can be tolerogenic [5, 7] or immunogenic [83], dictated by the microenvironment [83–85]. Those conditions should be crucial for the development of novel therapeutic approaches using CD103+ DCs in triggering mucosal immunity or tolerance.

Under steady-state conditions, lamina propria-resident CD103+ DCs are tolerogenic. However, inflammation induces MLN CD103+ DCs into a proinflammatory phenotype. For instance, MLN CD103+ DCs purified from colitic mice triggered Th1 responses along with high levels of IL6 production [83, 86]. During intestinal inflammation, MLN CD103+ DC acquires these proinflammatory properties with no phenotypical and ontogenetic changes.

Naturally occurring CD4+CD25+Foxp3+ Treg cells are thymus-derived and are important to modulate a wide range of immune-mediated pathologies, including autoimmunity, colitis, and chronic infection. However, inducible Treg cells arising from the naïve pool are particularly beneficial in the intestine. The balance of triggering protective immunity to invading pathogens while retaining tolerance to dietary antigen and the commensal flora is critical. These cells can be generated in the periphery from the naive T cell pool after, for example, the oral administration of antigen or the targeting of peptide ligands to DCs in vivo [87].

Some specific nutrients are known to have notable effects on the modulation of mucosal immunity. Moreover, mucosal DCs are constantly exposed to dietary antigens. Vitamin A, whose only source in mammals is through the diet, mediates several functions of CD103+ DCs. Its depletion from the diet inhibits Treg differentiation induced by MLN CD103+ DCs as well as inducing gut-homing receptors on T cells [88, 89].

Tryptophan is another example of dietary element that is required for the IDO-dependent tolerogenic effects of mucosal DCs [90] and for generation of ligands of the aryl hydrocarbon receptor (AhR), such as L-kynurenine that regulates the balance between Th17 and Treg cell differentiation [91–93] and has powerful direct anti-inflammatory activity on DCs [94].

Diet-derived lipid mediators can activate anti-inflammatory peroxisome proliferator-activated receptor (PPAR)*γ* [95]. Short chain fatty acids (including acetate, butyrate, and propionate) are among the most abundant metabolites derived from microbiota-mediated digestion of dietary fiber [96]. Exposure of monocyte-derived DCs to butyrate and propionate prevented proinflammatory cytokine release induced after LPS incubation [97]. In fact, animals deficient for butyrate receptor, GPR109a, are susceptible to the development of colitis and colon cancer [98].

Curcumin is a spice historically used as a medicine in India and Southeast Asia. Exposure of curcumin triggers a tolerogenic activity in DCs, including upregulation of* aldh1a2* and IL10 while promoting FoxP3+ Treg cells [99].

The mucosal neural anatomy is disrupted in inflammatory bowel diseases [100]; intestine is permeated by a complex nervous system. On the other hand, hematopoietic cells are responsive to neurotransmitters and mediators from the enteric nervous system exert immune-regulatory effects [100]. Vasoactive intestinal peptide (VIP) is produced by intestinal enteroendocrine and immune cells and has vasodilator and regulator of epithelial permeability activities [101]. VIP suppresses lipopolysaccharide-induced DC maturation [102] while promoting differentiation of IL10- and TGF-*β*-secreting Treg cells [103–105]. In agreement with these observations, DCs exposed to VIP to prevent chemically induced colitis [105]. Taken together, these studies revised here point to the complexity of interactions between mucosal DCs, nonimmune cells, the microbiota, and ingested nutrients. All these factors contribute to promoting and maintaining tolerance mediated by intestinal DCs under steady-state conditions. While no single mediator plays a dominant role, redundancy among several pathways and components is evolutionary advantageous to ensure that homeostasis is maintained.
